# Kinematic differences between left- and right-handed cricket fast bowlers during the bowling action

**DOI:** 10.17159/2078-516X/2023/v35i1a15144

**Published:** 2023-06-05

**Authors:** B Olivier, N Boulle, J Jacobs, OL Obiora, C MacMillan, J Liebenberg, S McErlain-Naylor

**Affiliations:** 1 Wits Cricket Research Hub for Science, Medicine and Rehabilitation, Department of Physiotherapy, School of Therapeutic Sciences, Faculty of Health Sciences, University of the Witwatersrand, Johannesburg, South Africa; 2 Sport Exercise Medicine and Lifestyle Institute (SEMLI), Faculty of Health Sciences, University of Pretoria, Pretoria, South Africa; 3 School of Sport, Exercise and Health Sciences, Loughborough University, United Kingdom

**Keywords:** biomechanics, dominance, handedness, laterality

## Abstract

**Background:**

Despite differences between left- and right-handed athletes in other sports, minimal evidence exists regarding biomechanical similarities and differences between left- and right-handed cricket fast bowlers performing an equivalent task.

**Objectives:**

This study aimed to compare the kinematics between left and right-handed fast bowlers performing an equivalent task (i.e. bowling ‘over the wicket’ to a batter of the same handedness as the bowler).

**Methods:**

Full body, three-dimensional kinematic data for six left-handed and 20 right-handed adolescent, male, fast bowlers were collected using the Xsens inertial measurement system. Time-normalised joint and segment angle time histories from back foot contact to follow-through ground contacts were compared between groups via statistical parametric mapping. Whole movement and subphase durations were also compared.

**Results:**

Left-handed players displayed significantly more trunk flexion from 49%–56% of the total movement (ball release occurred at 54%; *p* = 0.037) and had shorter back foot contact durations on average (0.153 *vs* 0.177 s; *p* = 0.036) compared to right-handed players.

**Conclusion:**

Left- and right-handed bowlers displayed similar sagittal plane kinematics but appeared to use non-sagittal plane movements differently around the time of ball release. The kinematic differences identified in this study can inform future research investigating the effect of hand dominance on bowling performance and injury risk.

Bowling is a critical aspect of cricket, where the bowler aims to restrict the runs scored by the opposing team. One way this is achieved is through fast bowling, i.e. maximising ball release speed and minimising batters’ response times. Fast bowlers and their coaches consistently seek enhanced ball release speeds via more effective biomechanical bowling actions.^[1, 2]^ For right-handed bowlers, this action consists of a run up to the bowling crease followed by landing on the right foot (referred to as back foot contact), stepping onto the left foot (front foot contact), and delivering the ball with the right hand (ball release) before following through onto the right foot again (follow through ground contact). The opposite is true for left-handed bowlers.

Laboratory-based (e.g. 3D motion capture ^[3]^), field-based (e.g. inertial measurement units [IMUs]^[4]^), and computer simulation^[2]^ approaches have been used to investigate associations between bowling technique parameters and performance or injury incidence. Each approach has advantages and limitations, with inertial measurement units enabling the measurement within an athlete’s habitual training environments and contexts. For example, Senington et al.^[5]^ used IMUs to investigate the relationship between spinal kinematics, lower limb accelerations, and ball release speed. Greater sacral vertical acceleration loading rate at back foot impact and thoracic side flexion at front foot impact was associated with faster ball release speeds. Similarly, IMUs have been used to establish the association between spinal kinematics, tibial impact, and low back pain in bowlers. Those without a history of low back pain had more thoracolumbar rotation between back and front foot contacts and greater times to peak tibial acceleration following front foot contact.^[4]^

Most investigations into fast bowling biomechanics have either explored the optimal technique for right-handed fast bowling^[2]^ or considered left- and right-handed bowlers within a single group.^[3]^ Kinematic differences have been reported between left- and right-handed players in other ball and bat sports, such as baseball pitching^[6]^ and tennis.^[7]^ Any overrepresentation of left-handedness in sports compared to the general public^[8]^ may be as a result of not only technical but also perceptual, tactical, or strategic factors.^[9]^ Knowledge of kinematic differences, such as those determined in this exploratory study, will form the basis of future research seeking to augment or counter the potential ‘unorthodox’ nature of the left-handed cricket bowling action. Therefore, descriptive studies make up an essential part of the knowledge base.

The aim of this study was therefore to compare the kinematics between left- and right-handed adolescent fast bowlers performing an equivalent bowling task. Due to the exploratory nature of this study and the lack of previous literature, no a priori hypotheses were made regarding the possible kinematic differences between the groups.

## Methods

### Study design and setting

This was a quantitative, cross-sectional study. Data collection took place at the cricket nets of the respective schools.

### Participants

Left- and right-handed injury free male schoolboy fast bowlers between the ages of 14 and 18 years participated in this study. All players played for their school’s cricket teams in a competitive high school league. Bowlers were classified by their coaches as fast bowlers following the accepted definition of a fast bowler where the wicketkeeper stands back from the stumps against them.^[10]^ The Human Ethics Research Committee (Medical) at the University of the Witwatersrand approved this study. All participants, and parents/caregivers of participants younger than 18 years, signed consent and assent forms, respectively, before data collection commenced.

### Instrumentation and outcome measures

Three-dimensional biomechanical data were collected using an Xsens inertial sensor motion analysis system (MVN Link Biomech, Xsens Technologies B.V., Enschede, Netherlands), a full-body human measurement system consisting of 17 IMUs recording at 240 Hz, biomechanical models, and sensor fusion algorithms. The IMUs were placed on the posterior head, sternum, pelvis (middle of the two posterior superior iliac spines, posterior shoulders (centre of scapulae), upper arms, forearms, posterior hands, lateral thighs, tibialis anterior and the superior feet. They were securely positioned within a Lycra bodysuit.^[11]^ Movement data were resolved via the MVN Analyze software into the respective segment and joint kinematic data. In addition to joint and segment angles, tri-axial acceleration was recorded via each IMU. Sagittal plane joint angles and non-sagittal torso-related angles from Xsens MVN have been compared against a 3D marker-based motion capture system during the analysis of gait and reported excellent wave similarity for sagittal angles and frontal torso-related angles, with very good to excellent similarity for transverse torso-related angles.^[12]^ Harnett et al^[13]^ investigated the outputs between an optical motion capture and an inertial measurement unit during bowling in cricket and found a mean difference of 4.7° in the shoulder girdle relative to pelvis angle and no difference between the two systems in terms of trunk and knee angles (mean differences being 0.1° and 1.6°, respectively). Cottam et al.^[14]^ stated that the differences between inertial measurement units and optical motion capture output might lie in the inherent errors associated with an optical motion capture system and confirmed that inertial measurement units are valid in the measurement of dynamic, multi-planar movements, such as the cricket fast bowling action.^[14]^

### Procedures

Anthropometric measurements of each participant were entered into Xsens software for calibration purposes. These measurements included body mass, stature, foot length, shoulder height and width, arm span, hip height and width, knee height, ankle height and sole height.

**Fig. 1:**
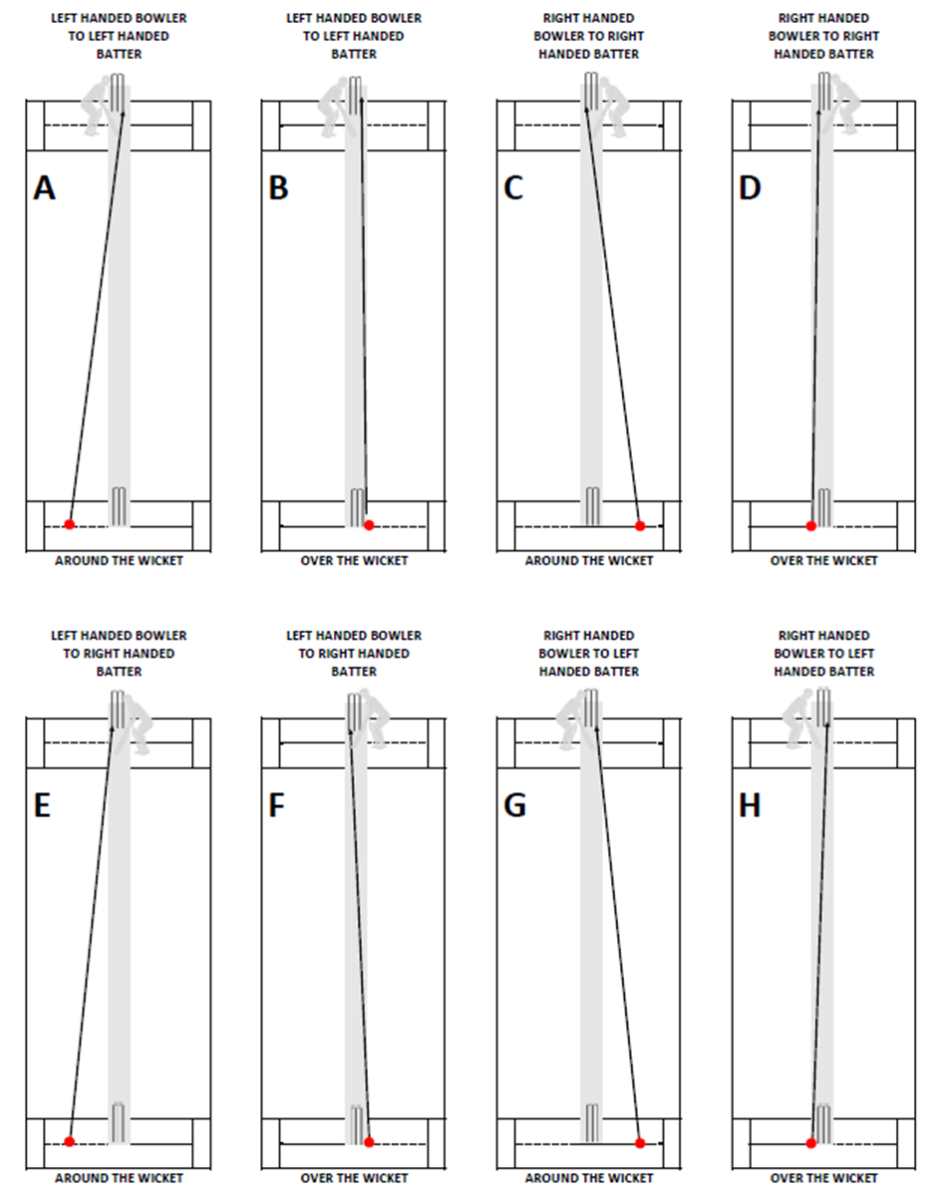
Angle of release for a left or right-handed bowler bowling ‘around’ or ‘over’ the wicket. (A) Left-handed bowler bowing around the wicket to a left-handed batter; (B) Left-handed bowler over the wicket to left-handed batter; (C) Right-handed bowler bowling around the wicket to a right-handed batter; (D) Right-handed bowler bowling over the wicket to a right-handed batter; (E) Left-handed bowler bowling around the wicket to a right-handed batter; (F) Left-handed bowler bowling over the wicket to a right-handed batter; (G) Right-handed bowler bowling around the wicket to a left-handed batter; (H) Right-handed bowler bowling over the wicket to a left-handed batter.

Each bowler performed a five-minute self-selected warm-up in their accustomed manner. Calibration was performed in the N-pose (arms neutral beside body) and during walking.^[11]^ Bowlers then bowled six match-paced deliveries using a 135g cricket ball. Bowlers were instructed to bowl 'over the wicket' (i.e. right-handed bowlers bowled from the left of the wicket to a right-handed batter (Figure 1D) and left-handed bowlers bowled from the right of the wicket to a left-handed batter (Figure 1B) towards the top of off stump. The angles of release for left and right-handed bowlers are shown in Figure 1.

Three-dimensional biomechanical data were recorded using the XSens Analyze software. The number of successful, analysed, trials was 4 ± 1 for the six left-handed bowlers and 4 ± 2 for 20 right-handed bowlers. Overall, 4 ± 2 trials were analysed for each of the 26 bowlers.

### Data processing

Front foot contact was identified as occurring three frames (0.013 s) before the peak resultant front tibial acceleration that occurred within 300 frames (1.25 s) prior to the overall (for the trial) peak resultant forearm acceleration. This was based on Lamb et al’s^[15]^ finding that peak resultant acceleration at the front tibia occurred on average 0.013 ± 0.006 s after front foot contact in cricket fast bowlers. Back foot contact was identified as occurring four frames (0.017 s) before the peak resultant back tibial acceleration that occurred within 100 frames (0.42 s) prior to front foot contact. This was based on Lamb et al’s^[15]^ finding that peak resultant acceleration at the back tibia occurred on average 0.016 ± 0.009 s after back foot contact in cricket fast bowlers. Ball release was identified as occurring at the instant of the peak resultant bowling forearm acceleration that was recorded within 50 frames (0.21 s) after front foot contact. This was based on Spratford et al’s^[16]^ use of peak outward wrist acceleration to successfully identify ball release within 0.014 s limits of agreement. The follow-through ground contact was identified as occurring five frames (0.021 s) before the peak resultant back tibial acceleration that occurred within 125 frames (0.52 s) after ball release. This was based on Lamb et al’s^[15]^ finding that peak resultant acceleration at the back tibia occurred on average 0.019 ± 0.011 s after the follow-through ground contact in cricket fast bowlers. The back foot contact phase was defined as beginning at back foot contact and ending at front foot contact. The front foot contact phase was defined as beginning at front foot contact and ending at ball release. The follow-through phase was defined as beginning at ball release and ending at the follow-through ground contact. The total movement was defined as the sum of these three phases.

For each bowler, the average duration of each of the three phases was determined as a percentage of the total movement. A weighted average of the entire sample, with left- and right-handed bowler groups weighted at 50% each, calculated the average durations to be 32% for back foot contact, 22% for front foot contact, and 46% for follow-through. All joint and segment angle data for each trial were time-normalised to 101 data points (0%–100% of the movement) via piecewise linear length normalisation.^[17]^ The back foot contact phase was normalised to 0%–32%, front foot contact 32%–54%, and follow-through from 54%–100% of the movement. All trials per player were ensemble averaged to produce a single time-normalised curve per player (i.e. six left-handed average curves and 20 right-handed average curves) for each front knee flexion-extension angle, back knee flexion-extension angle, front hip flexion-extension angle, back hip flexion-extension angle, pelvis transverse plane rotation, trunk transverse plane rotation, and trunk side flexion. All frontal and transverse plane angles for left-handed bowlers were adjusted (multiplied by −1) so that the anatomical definitions aligned to those used for right-handed bowlers could be directly compared.

### Statistical analysis

The distribution of phase and movement duration data was assessed via the Shapiro-Wilks’ test, with left-handers’ total movement (*W* = 0.725, *p* = 0.011) and back foot contact (*W* = 0.694, *p* = 0.005) durations, but no other durations (0.915 ≤ *W* ≤ 0.986, 0.065 < *p* < 0.985) deviating significantly from normality. Equality of variance was assessed via Levene’s test, with front foot contact (*F*(1) = 4.357, *p* = 0.048) but no other durations (0.926 ≤ *F*(1) ≤ 1.719, 0.202 ≤ *p* ≤ 0.345) deviating significantly from equal variance. Absolute durations (in seconds) were compared between left- and right-handed bowler groups via independent samples t-tests (parametric, for follow-through) or the Mann-Whitney test (non-parametric, for other durations) within JASP (v 0.16.2.0, Amsterdam, Netherlands). Data were reported as median (interquartile range) for all durations to enable direct comparisons. Estimates of effect size (ES, Cohen's *d* for t-test and rank biserial correlation for the Mann-Whitney test) and their 95% confidence intervals were reported. Effect sizes were interpreted as 0.1 ≤ small < 0.3, 0.3 ≤ medium < 0.5, and large ≥ 0.5.^[18]^ All time-normalised joint and segment angle one-dimensional waveforms were compared between left- and right-handed bowler groups via statistical parametric mapping independent samples t-tests using open source (https://www.spm1d.org) MATLAB (v 2022b, MathWorks, Natick, MA) script. For each continuous one-dimensional test, the critical test statistic and supra-threshold cluster were reported where the test statistic field exceeded the critical threshold. Alpha was set *a priori* at 0.05 for all discrete and continuous tests, with no control for multiple comparisons made due to the exploratory and hypothesis-generating nature of the study.

## Results

### Participant characteristics

Twenty-six injury free fast bowlers (6 left-handed, 20 right-handed) with a mean age of 15.4 ± 0.9 years participated in this study. Participants had a body height of 178.8 ± 5.2 cm, body mass 71.6 ± 8.1 kg, and body mass index of 22.4 ± 2.4 kg/m^2^.

### Movement durations

**Fig. 2:**
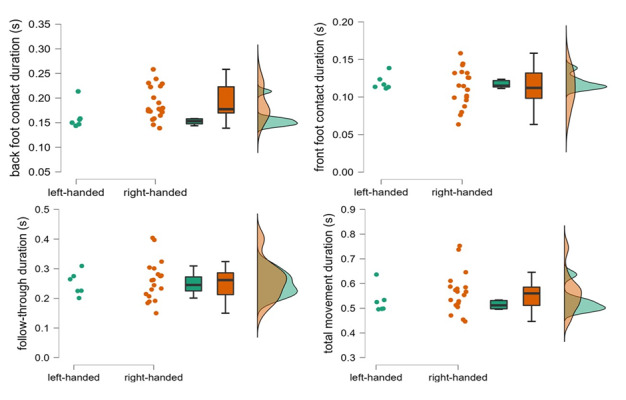
Distribution and individual data points for back foot contact phase (top left), front foot contact phase (top right), follow-through phase (bottom left), and total movement (bottom right) durations by left- and right-handed cricket fast bowlers. Horizontal lines on the box and whisker plots represent median and interquartile range.

Left-handed bowlers had significantly shorter absolute back foot contact durations (in seconds) than their right-handed counterparts (ES = 0.58 [95% CI: 0.13 – 0.84]; p = 0.036). Durations of other phases and the total movement were not significantly different between the groups (0.273 ≤ *p* ≤ 0.725; Table 1; Figure 2).

### Bowling kinematics

**Fig. 3:**
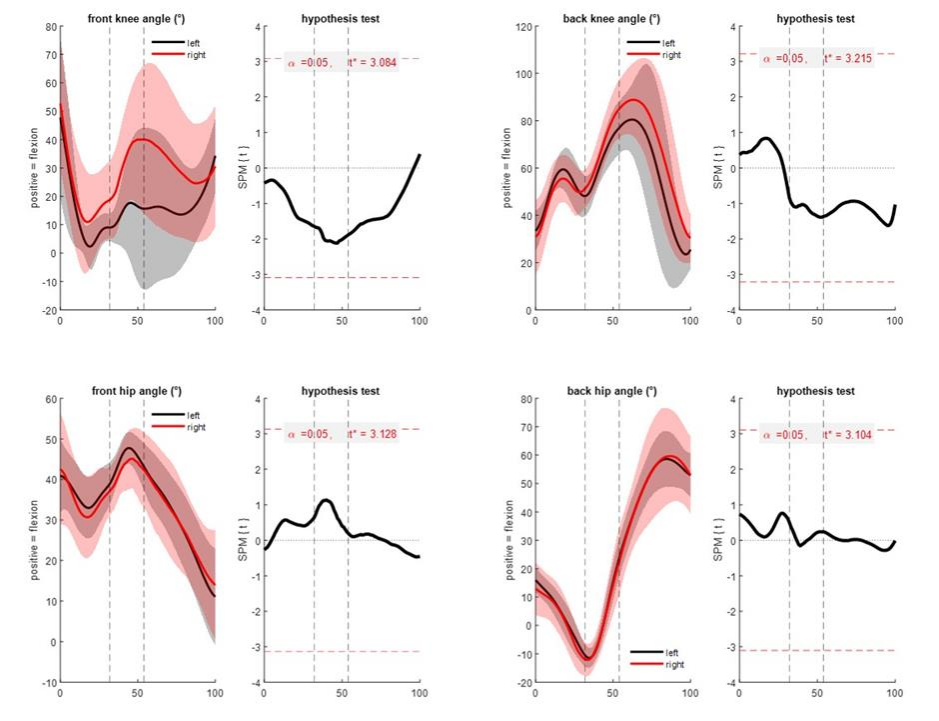
Sagittal plane joint angles: front knee flexion/extension (top left); back knee flexion/extension (top right); front hip flexion/extension (bottom left); and back hip flexion/extension (bottom right). Mean (solid lines) ± standard deviation (shaded areas) (left of each sub-figure) and statistical parametric mapping independent samples t-test result (right of each sub-figure) comparing left- and right-handed cricket fast bowlers from 0%–100% of the total time-normalised movement (back foot contact phase + front foot contact phase + follow-through phase, with individual phases separated by dashed vertical lines at front foot contact (left) and ball release (right)). The right-hand aspect of each sub-figure indicates statistical significance if the black t- statistic crosses the red dashed critical threshold.

**Fig. 4:**
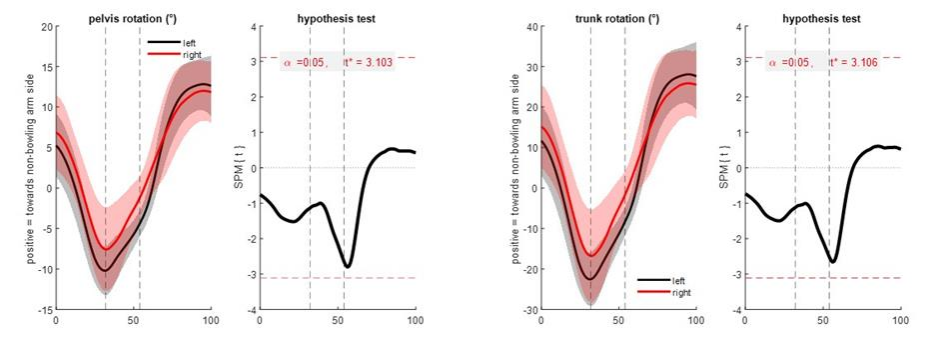
Transverse plane segment angles: pelvis rotation (left) and trunk rotation (right). Mean (solid lines) ± standard deviation (shaded areas) (left of each sub-figure) and statistical parametric mapping independent samples t-test result (right of each sub- figure) comparing left- and right-handed cricket fast bowlers from 0%–100% of the total time-normalised movement (back foot contact phase + front foot contact phase + follow-through phase, with individual phases separated by dashed vertical lines at front foot contact (left) and ball release (right). The right-hand aspect of each sub-figure indicates statistical significance if the black t- statistic crosses the red dashed critical threshold.

**Fig. 5:**
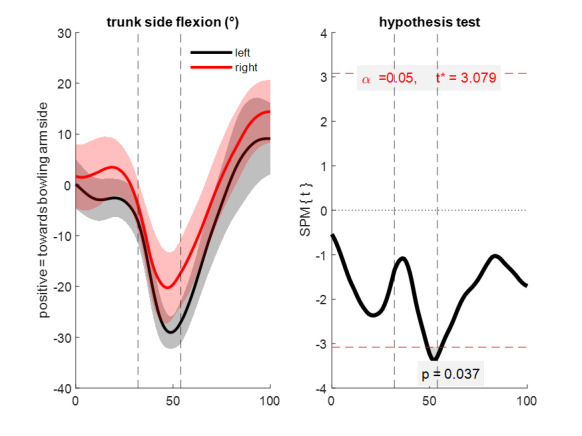
Frontal plane trunk side flexion angle. Mean (solid line) ± standard deviation (shaded area) (left) and statistical parametric mapping independent samples t-test result (right) comparing left- and right-handed cricket fast bowlers from 0%–100% of the total time-normalised movement (back foot contact phase + front foot contact phase + follow-through phase, with individual phases separated by dashed vertical lines at front foot contact (left) and ball release (right)). The right-hand graph indicates statistical significance if the black t-statistic crosses the red dashed critical threshold.

There were no significant differences between left- and right-handed bowlers in any measured sagittal plane (flexion/extension) angles at any time in the movement (Figure 3). In the transverse plane (Figure 4), although there were again no significant differences (p > 0.05), peak differences in mean pelvis (3.3°; left > right) and trunk (6.8°; left > right) rotation occurred close to ball release timing (56% of the movement, with ball release at 54%). In the frontal plane (Figure 5), left-handed players had significantly more trunk side flexion (p = 0.037, peak difference 9.8°) during the final part of the front foot contact phase and slightly after ball release (from 49 – 56% of the movement) compared to right-handed bowlers.

## Discussion

The current study sought to determine whether there are kinematic differences between left- and right-handed fast bowlers and to consequently contribute to the limited research on left-handed bowlers. The main findings when comparing left- and right-handed bowlers in this study were that left-handed bowlers spent less time at back foot contact and utilised more trunk-side flexion. They also had qualitatively, but not significantly, greater pelvis and trunk longitudinal rotations.

The left-handed bowlers utilised more trunk side flexion than the right-handed bowlers. Unfortunately, increased trunk side flexion has also been linked to a higher risk of sustaining a lower back injury.^[19, 20]^ The posterior muscles within the lumbopelvic region play a prominent role in stabilising the spine during the bowling action, specifically when compressive and shear forces are high.^[21]^ Protective morphological abdominal wall muscle adaptations have also been described in studies investigating risk factors related to lower back injuries among pace bowlers.^[22, 23]^ Implementation of strategies aimed at activating posterior lumbopelvic and abdominal wall muscles to ultimately offset forces related to increased trunk side flexion might therefore be especially relevant to left-handed bowlers. However, future research is needed to confirm this hypothesis. Determining the relationship between hand dominance and lower back injury risk is beyond the scope of our study in that our inclusion criteria required bowlers to be injury free and the cross-sectional descriptive study design did not allow for causality to be established.

The increase in trunk side flexion and possible but unclear increases in trunk rotations may have been facilitated by an earlier grounding of the front foot, resulting in shorter back foot contact phases in left-handed bowlers within this study. While it appeared that one left-handed player had longer movement phase durations compared to other left-handed players (Figure 2), it is important to note that this was not consistent across all phases and not necessarily the same player. Due to this study’s exploratory nature, a relatively small sample size was employed and although it seems as if the parameters were mechanically related to one another, this is a hypothesis worthy of exploration in future studies. Human movement is complex, and it is important to consider the integration of the various movement components throughout the entire movement.

This study required left-handed bowlers to bowl over the wicket to a left-handed batter; however, this is not a common occurrence in cricket. Left-handed bowlers may have adapted their bowling technique because of frequently bowling to right-handed batters. The majority of batters use a right-handed batting technique. Brooks et al^[24]^ found that only 24% of batters in the 2003 cricket World Cup were left-handed. Furthermore, considering that only 8% of fast bowlers are left-handed^[25]^ the chances are very slim that a left-handed fast bowler will bowl to a left-handed batter. When a left-handed bowler bowls to a left-handed batter, these movement components which developed when bowling to a right-handed batter, may have remained ingrained in their bowling actions.

The unique kinematic strategies displayed by left-handed bowlers when compared to right-handed bowlers seemed to be motivated by a deliberate in-game strategic approach. A right-handed bowler bowling over the wicket to a right-handed batter and aiming for the ball to hit the top of the off stump (as shown in Figure 1D), will bowl in a fairly straight line. However, when a left-handed bowler bowls over the wicket to a right-handed batter (Figure 1F), in aiming for the top of the off stump, and to avoid the danger area on the pitch, the left-handed bowler needs to bowl at an angle. It is therefore likely that the left-handed bowlers employed more trunk side flexion and possibly rotation to achieve their goal.

All bowlers were given an equivalent bowling task in that they bowled ‘over the wicket’ towards a batter of the same handedness as they are (Figures 1B and D). The left- and right-handed bowlers therefore performed a bowling task as a mirror image of one another. When tasked with bowling from the left of the wicket to a right-handed batter, left-handed bowlers will necessarily be releasing the ball from a relatively wider release position (‘around the wicket’ as shown in Figure 1E) compared to their right-handed counterparts performing the same task (‘over the wicket’ as shown in Figure 1H). Bowling ‘around the wicket’ compared to ‘over the wicket’ may lead to differences in bowling kinematics. The instructions given to bowlers in terms of the above should be taken into consideration in the methods of future studies. Although the approach taken in our study ensured comparability of bowling technique, it was also an unnatural situation for a left-handed bowler to bowl to a left-handed batter seeing that there are very few left-handed batters.

A limitation of this study is that an aspect of temporal uncertainty will have been introduced by the estimation of ground contact and ball release events informed by peak resultant accelerations and literature values. The literature values used to inform these estimations reported ground contact standard deviations of 0.006–0.011 s^[15]^ and ball release limits of agreement of 0.014 s.^[16]^ This limitation relates to the commonly encountered trade-off between field- and laboratory-based data collection methodologies.

Considering the exploratory, descriptive nature of this cross-sectional study, no practical recommendations can be made to coaches, players and clinicians at this stage. The findings from this study will inform future research investigating the potential to augment or counter the potential ‘unorthodox’ nature of the left-handed cricket bowling action. For example, knowledge of the increased trunk side-flexion in left-handed bowlers may inform research into coaching interventions specific to variations in the bowling task. In addition, future studies exploring the role of handedness in injury risk given the known links between trunk kinematics and lumbar injuries in cricket fast bowlers will add further value in terms of clinical implications.

## Conclusion

Left- and right-handed bowlers displayed similar sagittal plane kinematics when performing an equivalent bowling task. However, they appeared to use non-sagittal plane movements differently around the time of ball release. Primarily, left-handed bowlers utilised more trunk side flexion. They also had shorter back foot contact durations on average compared to right-handed players. The kinematic differences identified in this study can inform future research investigating the effect of hand dominance on bowling performance and injury risk.

## Figures and Tables

**Table 1:** Median (interquartile range) durations of total movement and individual phases for left- and right-handed cricket fast bowlers

Movement phase	Left-handed (s)	Right-handed (s)	p-value	Effect size	95% confidence interval
Back foot contact	0.153 (0.148 – 0.158)	0.177 (0.170 – 0.223)	0.036	0.58	0.13 – 0.84
Front foot contact	0.115 (0.113 – 0.122)	0.112 (0.098 – 0.132)	0.523	0.18	−0.33 – 0.62
Follow-through	0.245 (0.225 – 0.272)	0.262 (0.213 – 0.286)	0.725	0.17	−0.75–1.08
**Total**	0.512 (0.498 – 0.531)	0.560 (0.511 – 0.585)	0.273	0.31	−0.22 – 0.69

Statistical tests correspond to independent samples t-test (Cohen's d effect size) for follow-through and the Mann-Whitney test (rank biserial correlation effect size) for other durations.
